# Inflammatory Potential of Diet and Breast Cancer Risk: A Population‐Based Case–Control Study

**DOI:** 10.1002/fsn3.72473

**Published:** 2026-10-13

**Authors:** Reza Amani‐Beni, Sherry Price, Bahar Darouei, Ibrahim Abdollahpour, Shaghayegh Haghjooy Javanmard, Kazem Zendehdel, James R. Hebert

**Affiliations:** ^1^ Isfahan Cardiovascular Research Center, Cardiovascular Research Institute Isfahan University of Medical Sciences Isfahan Iran; ^2^ Cancer Prevention and Control Program and Department of Epidemiology and Biostatistics, Arnold School of Public Health University of South Carolina Columbia South Carolina USA; ^3^ Department of Nutrition Connecting Health Innovations LLC (CHI) Columbia South Carolina USA; ^4^ Anesthesiology and Critical Care Research Center Isfahan University of Medical Sciences Isfahan Iran; ^5^ Research Institute for Primordial Prevention of Non‐Communicable Disease Isfahan University of Medical Sciences Isfahan Iran; ^6^ Metabolomics and Genomics Research Center, Cellular and Molecular Institute, Endocrinology and Metabolism Research Institute Tehran University of Medical Sciences Tehran Iran; ^7^ Cancer Biology Research Center, Cancer Institute Tehran University of Medical Sciences Tehran Iran

**Keywords:** breast cancer, case–control studies, dietary patterns, energy intake, inflammation

## Abstract

Chronic inflammation may contribute to breast cancer (BC). The Dietary Inflammatory Index (DII) and energy‐adjusted DII (E‐DII) quantify the inflammatory potential of diet. This case–control study evaluated associations of the DII and E‐DII with BC risk among Iranian women. We recruited 600 newly diagnosed BC cases and 600 population‐based controls from Isfahan, Iran. Dietary intake was assessed using a validated 168‐item food frequency questionnaire (FFQ). DII and E‐DII scores were calculated based on 26 and 25 food parameters, respectively. Logistic regression models were used to estimate odds ratios (ORs) and 95% confidence intervals (CIs). Multivariable‐adjusted models accounted for confounders including age, body mass index, reproductive history, and sunlight exposure. Higher E‐DII scores were significantly associated with an increased odds of developing BC. In the fully adjusted models, women in the highest quartile of the E‐DII had 63% greater risk than those in the lowest quartile (adjusted OR: 1.63; 95% CI: 1.08–2.45). Analyses based on continuous E‐DII also showed that each unit increase in the E‐DII score was linked to a 14% increase in BC odds (adjusted OR: 1.14; 95% CI: 1.05–1.24). In contrast, DII scores were not significantly associated with BC risk in any of the models. This study demonstrated that diets with stronger inflammatory properties, indicated by elevated E‐DII scores, are associated with a higher risk of BC among Iranian women. These outcomes underline the value of using energy‐normalized inflammatory indices when assessing dietary influences on cancer risk.

## Graphical abstract

In a case–control study of 600 breast cancer cases and 600 controls from Isfahan, Iran, a more pro‐inflammatory diet, measured by the energy‐adjusted Dietary Inflammatory Index (E‐DII), was associated with higher breast cancer risk (adjusted OR 1.63, highest vs. lowest quartile).

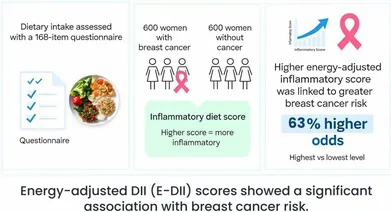

## 1 Introduction

Globally, breast cancer (BC) is the most prevalent cancer in women and a leading cause of cancer‐related mortality (Aggarwal and Bagri 2025). In 2021, over 2.1 million women were diagnosed worldwide, and BC accounted for more than 674,000 deaths (Sha et al. 2024). In Iran, despite advances in early detection and treatment globally, the age‐standardized BC mortality rate increased by 16.5% from 1990 to 2019 (Darbandi et al. 2025). Recent national health insurance data similarly show rising mortality from 0.68 to 0.86 per 100,000 individuals between 2021 and 2022 (Seyed‐Nezhad et al. 2025). Iranian women are also commonly diagnosed about a decade earlier and often at more advanced stages than women in Western countries (Kolahdoozan et al. 2010).

Beyond reproductive, hormonal, and genetic determinants (Faupel‐Badger et al. 2013; Clendenen et al. 2013), chronic inflammation is a plausible contributor to BC development (Summer et al. 2024). Inflammatory pathways may promote carcinogenesis through cytokine release and downstream effects, reactive oxygen species generation, DNA damage, and sustained signaling that supports tumor initiation and progression (Summer et al. 2024). Diet is a key modulator of systemic inflammation and may either attenuate or amplify these processes (Wagenaar et al. 2021).

To quantify the inflammatory potential of diet, the Dietary Inflammatory Index (DII) (Shivappa et al. 2014) and its energy‐adjusted version (E‐DII) were developed (Hébert et al. 2019). These indices have been validated through associations with inflammatory biomarkers in 63 studies and studied extensively across cancer outcomes in hundreds of studies (Wang et al. 2026; Bakhshimoghaddam et al. 2026; Nikparast et al. 2025; Dai et al. 2024). Evidence suggests that more pro‐inflammatory diets, reflected by higher DII/E‐DII scores, may increase BC risk (Huang et al. 2017; Obón‐Santacana et al. 2019), although null findings have also been reported (Gardeazabal et al. 2019; Hammad et al. 2021). Given regionally distinct dietary patterns, we conducted a population‐based incident case–control study to examine the associations of DII and E‐DII with BC in Iranian women, designed to assess whether energy adjustment improves risk estimation in this setting.

## 2 Methods and Materials

This population‐based case–control study was conducted from May 2021 to October 2023 in Isfahan, Iran (Abdollahpour et al. 2025; Darouei et al. 2025). BC patients were identified from the Isfahan Breast Cancer Registry (Roshandel et al. 2019) and recruited if they had histologically confirmed, newly diagnosed BC with no previous cancer history. Of the 653 newly identified cases during the study period, 600 (92%) agreed to participate.

Controls were women aged 18–75 years residing in the 15 municipal districts of Isfahan, who had no history of BC. To identify eligible controls, we applied a standard random digit dialing (RDD) approach (Wacholder et al. 1992) using the same source population as the BC cases (approximately 780,000 women). Of the 852 eligible households identified by the RDD process, 252 declined to participate (101 refused to participate in the initial screening interview and 151 declined participation in the main study interview). Finally, 600 eligible women identified through the RDD completed the main study questionnaire, yielding a response rate of 70.4% (Figure 1).

**FIGURE 1:**
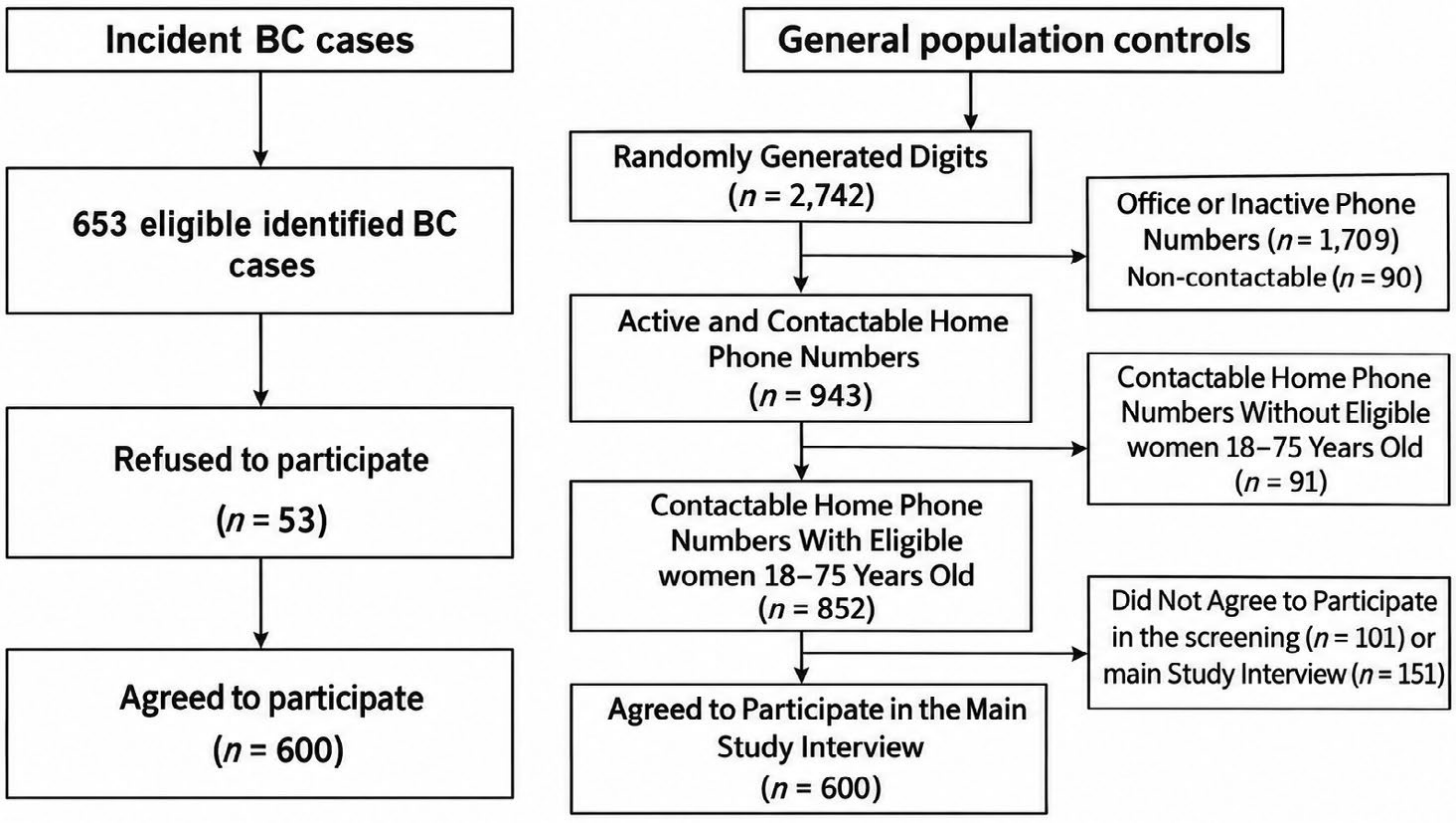
Recruitment flowchart of BC incident cases and general population women controls for the study of the association between DII and breast cancer risk, Isfahan, Iran, 2021–2023.

### 2.1 Data Collection Protocol

Five trained female interviewers, selected for their communication and interviewing skills, conducted structured telephone interviews. To ensure data quality and minimize interviewer bias, interviews were randomly recorded and reviewed as part of the monitoring process. Considering the phone interviews method used, verbal informed consent was obtained from all participants prior to enrollment. The study was approved by the Ethics Committee of Isfahan University of Medical Sciences (IR.MUI.REC.1399.010).

### 2.2 Calculation of DII and E‐DII Scores

Dietary intake was assessed using a validated 168‐item Food Frequency Questionnaire (FFQ) (Mirmiran et al. 2010). Using standard household measures, portion sizes were converted to grams per day (Ghaffarpour et al. 1999). Nutrient and energy intake data were processed using the Nutritionist IV software adapted to Iranian foods. The inflammatory potential of participants' diets was assessed using the DII and E‐DII (Shivappa et al. 2014; Hébert et al. 2019).

DII scores were calculated using 35 of the 45 original food parameters (Abdollahpour et al. 2020), including macronutrients, fatty acids, cholesterol, fiber, and selected vitamins and minerals. For each participant, nutrient intakes were standardized against a global reference database to generate *z*‐scores, converted to centered percentiles, and multiplied by literature‐derived inflammatory effect scores. The resulting values were summed to obtain an overall DII score, with higher scores indicating a more pro‐inflammatory diet.

E‐DII scores were calculated using the same components and scoring algorithm based on 34 available components, except that nutrient intakes were first adjusted for total energy intake and expressed per 1000 kcal. This approach isolates dietary inflammatory potential from overall caloric intake and improves comparability across individuals. The energy‐adjusted global reference database was applied in accordance with international standards. Shown in Table S1 are the 35 parameters used for DII calculation and the 34 parameters used for E‐DII calculation (total energy was in the denominator).

We also obtained information on age, years of education, age at menarche, menopausal status, lifetime alcohol intake, history of hormone use including oral contraceptives (OCP), and menstrual bleeding duration, average lifetime sunlight exposure, and marital status. Height and weight were self‐reported and used to calculate the body mass index (BMI) as weight (kg) divided by height squared (m^2^) (Amani‐Beni et al. 2023).

### 2.3 Statistical Analysis

The crude associations between DII and E‐DII, and BC risk were estimated using univariate logistic regression models. Multivariable logistic regression models, with BC as the outcome, were then employed to estimate the odds ratios (ORs) and 95% confidence intervals (CIs) of the DII and E‐DII, adjusted for potential confounders. The DII and E‐DII were analyzed as continuous variables and categorized into quartiles, and as binary variables (≥ median and < median). Simple logistic regression was performed to estimate crude ORs. Adjustments were made for potential confounders, such as age, years of education, menopausal status, menarche age, marital status, BMI, physical activity, lifetime alcohol intake, history of hormone use including OCP, menstrual bleeding duration and average lifetime sunlight exposure during summer. Linear trends across quartiles were evaluated by modeling the median values of each quartile as a continuous term. We used the Hosmer–Lemeshow test to assess the goodness‐of‐fit of all multivariable logistic models. All statistical analyses were performed using STATA version 12. Statistical significance was defined as *p* < 0.05. Selection of the DII or E‐DII in statistical models was based on model goodness‐of‐fit.

## 3 Results

A total of 600 newly diagnosed BC cases and 600 controls from the general population were included in this study (Table 1). Compared to controls, cases were more likely to be older, postmenopausal (54.4% vs. 35.2%; *p* < 0.001), and have a higher average education level (12.4 vs. 9.5 years; *p* < 0.001). Earlier age at menarche was also more prevalent in these cases (13.2 vs. 13.5 years; *p* = 0.001). No significant differences were observed in BMI or sunlight exposure.

**TABLE 1:** Characteristics of 600 breast cancer cases and 600 general population controls, Isfahan, Iran, 2021–2023.

Variables	Case (*n* = 600)	Control (*n* = 600)	Crude OR, 95% CI	*p*
Age (years); mean (SD)	50.0 (9.7)	45.9 (12.9)	1.03 (1.02, 1.04)	0.001
Education (years)	12.39 (4.47)	9.52 (5.05)	1.13 (1.10, 1.16)	< 0.001
Menopause				
Yes	327 (54.40)	211 (35.17)	2.21 (1.75, 2.78)	< 0.001
Menarche age (years)	13.23 (1.73)	13.55 (1.67)	0.89 (0.84, 0.96)	0.001
Marital status				
Married	480 (80.13)	470 (78.46)	1.11 (0.84, 1.46)	0.48
Body mass index (kg/m^2^); mean (SD)	27.10 (4.81)	26.76 (4.63)	1.01 (0.99, 1.04)	0.22
Average lifetime summer sunlight exposure (hours per day)	1.42 (1.34)	1.55 (1.27)	0.92 (0.84, 1.01)	0.09
Energy intake	1728 (876)	1717 (1026)	0.999 (0.999, 1.00)	0.85

### 3.1 Association Between E‐DII and Breast Cancer

The mean (SD) E‐DII was higher among cases 1.77 (1.90) than in controls 1.63 (1.69) (Table 2). In the unadjusted model, per‐unit E‐DII score was not significantly associated with the odds of BC (OR = 1.04; 95% CI: 0.98–1.11; *p* = 0.17). However, a significant association was observed after adjusting for the potential confounders. Each one‐unit increase in E‐DII was associated with 14% higher odds of BC (OR = 1.14; 95% CI: 1.05–1.24; *p* = 0.002). Analyses based on median split were marginally significant (OR = 1.21 (0.91, 1.61); *p* = 0.10). When categorized into quartiles, those in the highest E‐DII quartile had significantly higher odds of developing BC than those in the lowest quartile (OR = 1.63; 95% CI: 1.08–2.45; *p* = 0.02). In addition, the linear trend across quartiles was statistically significant (*p*_trend_ = 0.04).

**TABLE 2:** Crude and adjusted association of DII and E‐DII with breast cancer odds.

	Case (*n* = 600)	Control (*n* = 600)	Crude OR (95% CI), *p*	Adjusted^a^ OR (95% CI), *p*
E‐DII (Continuous)	1.77 (1.90)	1.63 (1.69)	1.04 (0.98, 1.11), 0.17	1.14 (1.05, 1.24), **0.002**
E‐DII				
≥ Median (1.44)	303 (50.5%)	297 (49.5%)	1.04 (0.83, 1.30), 0.17	1.21 (0.91, 1.61), 0.10
E‐DII (Quartiles)				
Q1: −2.19, 0.32	158 (26.33)	142 (23.67)	References	References
Q2: 0.33, 1.44	139 (23.17)	161 (26.83)	0.77 (0.56, 1.07), 0.12	1.11 (0.74, 1.65), 0.61
Q3: 1.45, 2.76	139 (23.17)	161 (26.83)	0.77 (0.56, 1.07), 0.12	1.02 (0.69, 1.52), 0.92
Q4: > 2.76	164 (27.33)	136 (22.67)	1.08 (0.79, 1.49), 0.62	1.63 (1.08, 2.45), **0.02**
Test for trend			1.02 (0.92, 1.13), 0.64	1.14 (1.01, 1.30), **0.04**
DII (Continuous)	2.72 (2.20)	2.61 (2.14)	1.02 (0.97, 1.08), 0.38	1.05 (0.98, 1.12), 0.15
DII				
≥ Median (2.89)	303 (50.5%)	297 (49.5%)	1.04 (0.83, 1.30), 0.73	1.08 (0.81, 1.43), 0.60
DII (Quartiles)				
Q1: −2.56–0.906	150 (25.00)	152 (25.33)	References	References
Q2: 0.907–2.89	147 (24.50)	151 (25.17)	0.99 (0.72, 1.35), 0.93	0.92 (0.62, 1.35), 0.67
Q3: 2.90–4.63	145 (24.17)	155 (25.83)	0.95 (0.69, 1.30), 0.74	0.88 (0.59, 1.31), 0.53
Q4: > 4.63	158 (26.33)	142 (23.67)	1.13 (0.82, 1.55), 0.46	1.23 (0.82, 1.86), 0.32
Test for trend			1.03 (0.93, 1.14), 0.54	1.06 (0.93, 1.20), 0.40

^a^ Adjusted for age, years of education, menopause status, menarche age, marital status, BMI, physical activity, lifetime alcohol intake, history of hormone use including OCP, menstrual bleeding duration and average lifetime sunlight exposure (summer).

### 3.2 Association Between DII and Breast Cancer

Although mean (SD) DII scores were higher among cases 2.72 (2.20) than controls 2.61 (2.14), the unadjusted and adjusted analyses of the DII did not reveal a statistically significant association with odds of BC (Table 2). The crude and adjusted OR per each unit increase in DII was OR = 1.02 (95% CI: 0.97–1.08; *p* = 0.38) and OR = 1.05 (95% CI: 0.98–1.12; *p* = 0.15). In the adjusted model, categorical analysis according to the DII quartiles also showed no significant difference in the odds of BC between the highest and lowest quartiles (OR = 1.23; 95% CI: 0.82–1.86; *p* = 0.32). The linear trend across the DII quartiles was also not statistically significant (*p* for trend = 0.40).

## 4 Discussion

Considering the inherent limitation of case–control studies, the results of our large population‐based case–control study revealed that, in both continuous and quartile analysis, a higher inflammatory diet characterized by E‐DII was associated with higher odds of BC in Iranian women. In the adjusted analysis, those in the highest E‐DII quartile had 63% greater odds of being diagnosed with BC compared to those in the lowest quartile. Although the direction of the association was consistent, the median‐split analysis was only marginally significant, indicating some sensitivity to E‐DII categorization. Because dichotomization may reduce information and, consequently, statistical power, these findings should be interpreted cautiously. In contrast, no statistically significant association was found between DII and risk of BC. These findings suggest that adjusting for energy intake may enhance the predictive capacity of dietary inflammatory potential in relation to BC risk.

Several earlier studies have reported a significant association between higher DII scores and increased BC risk (Huang et al. 2017; Hajji‐Louati et al. 2023; Shivappa et al. 2017; Jalali et al. 2018; Hayati et al. 2022). However, our findings are in line with those of studies that found no association between DII and BC risk (Gardeazabal et al. 2019; Ge et al. 2015; Tabung et al. 2016). Nevertheless, the broader literature supports a link between DII and BC. According to a meta‐analysis by Chen et al. individuals with elevated DII values faced a significantly higher risk of developing BC (relative risk = 1.37) (Chen et al. 2021). These inconsistencies may reflect the multifactorial nature of BC, in which the interplay of several risk factors complicates efforts to isolate the independent effects of diet (Faupel‐Badger et al. 2013; Hammad et al. 2021) as well as the importance of controlling for energy intake (Hébert et al. 2019).

Many studies have also examined the role of E‐DII in BC development. Similar to the DII, there are inconsistencies in findings in previous studies. In a large‐scale case–control study in northwestern Iran, Hayati et al. identified a strong positive link between elevated E‐DII scores and an 87% higher risk of BC (Hayati et al. 2022). In contrast, Obón‐Santacana et al.'s research failed to find a statistically significant link between E‐DII values and BC incidence, with a non‐significant positive trend in a population of Spanish women (Obón‐Santacana et al. 2019). Park et al. found that E‐DII was significantly associated only with triple‐negative BC, but not with BC overall, highlighting its potential relevance for specific subtypes (Park et al. 2021).

Differences between DII and E‐DII associations have been reported previously (DiNatale et al. 2023). DII scores are influenced by total food intake, whereas energy consumption generally declines with age (Hébert et al. 2019; Giezenaar et al. 2016). Variability in findings may also reflect individual differences in inflammatory responses and the diverse nutritional composition of foods included in DII calculations (Huang et al. 2017; Shivappa et al. 2017, 2015; Ge et al. 2015). Therefore, adjusting for energy intake is essential when evaluating dietary inflammatory potential across populations (DiNatale et al. 2023). Consistent evidence linking higher DII scores with lower energy intake led to the development of the E‐DII (Ramallal et al. 2017; Ruiz‐Canela et al. 2015; Alkerwi et al. 2015). Our findings support this approach, as E‐DII better captures inflammatory nutrient density independent of total caloric intake.

Our findings are biologically plausible, as chronic inflammation plays a key role in breast cancer development (Coussens and Werb 2002). Pro‐inflammatory cytokines, including IL‐6, TNF‐α, and IL‐1β, are elevated in patients with BC and have been associated with poorer prognosis (Mazidi et al. 2017; Jodi and Kathryn 2010). Notably, IL‐6 promotes tumor progression through activation of the STAT3 signaling pathway, enhancing cancer cell migration and invasiveness (Dethlefsen et al. 2013). Diet is a major modulator of systemic inflammation; saturated fats and refined carbohydrates promote inflammation, whereas fiber and other bioactive compounds exert anti‐inflammatory effects (Chen et al. 2021). Natural compounds characteristic of the Mediterranean diet, particularly polyphenols from extra‐virgin olive oil, fruits, vegetables, and nuts, may help protect against BC through antioxidant, anti‐inflammatory, antiproliferative, and pro‐apoptotic effects (Perrone et al. 2025). These mechanisms support the utility of DII and E‐DII as measures of dietary inflammatory potential.

The strengths of this study include the use of random sampling to recruit population‐based controls, a relatively large sample size, and high response rates among newly diagnosed BC cases. Nevertheless, several limitations should be considered. First, because of the case–control design, temporal relationships and cause‐and‐effect inferences cannot be established. Second, retrospective dietary assessment may have introduced measurement error, and recall bias is an inherent limitation of case–control studies. The direction and magnitude of this bias depend on whether misclassification is differential or non‐differential and on the nature of measurement errors in both exposure and confounding variables (Rothman 2008). Furthermore, the FFQ relied on self‐reported dietary intake and may have been affected by difficulties in estimating portion sizes, omission of snacks, and underestimation of total energy intake. Self‐reported height and weight may also have been subject to reporting error. Third, only 35 of the 45 original DII components and 34 corresponding E‐DII components were available, which may have reduced the precision of DII and E‐DII estimates. Fourth, residual confounding, including potential genetic influences, parity, breastfeeding history, and smoking, cannot be ruled out. Fifth, recruitment of controls through RDD limited participation to individuals with residential landlines, potentially underrepresenting some segments of the target population and introducing selection bias (Wacholder et al. 1992). However, the high response rates among both cases and controls likely minimized the impact of such bias. Finally, no formal adjustment for multiple comparisons was made, as the six models represented different operationalizations of the same underlying exposure rather than independent hypothesis tests; therefore, the consistency of findings across the different operationalizations of DII/E‐DII should be interpreted with this in mind.

## 5 Conclusion

Despite the limitations of case–control studies, our findings suggest that a pro‐inflammatory diet, assessed using the E‐DII, is associated with higher odds of breast cancer. The stronger association observed for E‐DII than DII underscores the importance of energy adjustment in nutritional epidemiology. These results provide hypothesis‐generating evidence regarding anti‐inflammatory dietary patterns rich in fruits, vegetables, and whole grains. Prospective and intervention studies are needed to confirm causality and evaluate preventive potential before specific public health recommendations can be made.

## Author Contributions

**Reza Amani‐Beni:** investigation, writing – original draft, formal analysis, writing – review and editing. **Sherry Price:** investigation, writing – original draft, formal analysis, data curation, writing – review and editing. **Bahar Darouei:** investigation, writing – review and editing, writing – original draft, data curation. **Ibrahim Abdollahpour:** conceptualization, investigation, funding acquisition, writing – original draft, methodology, validation, visualization, writing – review and editing, project administration, data curation, supervision. **Shaghayegh Haghjooy Javanmard:** conceptualization, investigation, writing – original draft, writing – review and editing, methodology, project administration. **Kazem Zendehdel:** conceptualization, investigation, writing – review and editing, methodology, supervision. **James R. Hebert:** investigation, writing – review and editing, validation, methodology, formal analysis, supervision.

## Funding

This work was supported by Isfahan University of Medical Sciences (grant number: 199061) and the Shams Charity Organization and Cancer Institute of Iran, Tehran University of Medical Sciences (grant number: 1400‐2‐244‐53323). James R. Hébert and Sherry Price were supported by the US National Cancer Institute‐funded REMEDY Study grant U01 CA272977‐01, which is part of the MeDOC Consortium. The content of this paper is solely the responsibility of the authors and does not necessarily represent the official views of the National Institutes of Health.

## Ethics Statement

Verbal informed consent was obtained from all participants. The ethics committee of the Isfahan University of Medical Sciences approved this study (IR.MUI.REC.1399.010).

## Consent

The authors have nothing to report.

## Conflicts of Interest

James R. Hébert discloses that he owns a controlling interest in Connecting Health Innovations LLC (CHI), which has licensed exclusive rights to the DII from the University of South Carolina and full ownership of the energy‐adjusted DII (E‐DII). CHI had no role in study design, data collection, data analysis, interpretation of results, manuscript preparation, or the decision to submit the work for publication. All other authors declare no competing interests.

## Supporting information


**Table S1:** Parameters used for DII and E‐DII calculation.

## Data Availability

The data that support the findings of this study are available on request from the corresponding author. The data are not publicly available due to privacy or ethical restrictions.
