# Supplementary material for: Inflammatory Potential of Diet and Breast Cancer Risk: A Population‐Based Case–Control Study
Source: Food Sci Nutr. 2026 Oct 13;14(10):e72473. doi: 10.1002/fsn3.72473 (PMC13660406; doi:10.1002/fsn3.72473)
Supplement: Supplementary file 1 [file FSN3-14-e72473-s001.docx]

**Supplementary Table 1. Parameters used for DII and E-DII calculation**

| **DII Parameter** | **Required Unit** |
| --- | --- |
| Energy | kcal/day |
| Carbohydrate | g/day |
| Protein | g/day |
| Total Fat | g/day |
| Fiber | g/day |
| Cholesterol | mg/day |
| Saturated Fat (SFA) | g/day |
| Monounsaturated Fat (MUFA) | g/day |
| Polyunsaturated Fat (PUFA) | g/day |
| Omega-3 | g/day |
| Omega-6 | g/day |
| Trans Fat | g/day |
| Niacin | mg/day |
| Thiamin | mg/day |
| Riboflavin | mg/day |
| Vitamin B12 | µg/day |
| Vitamin B6 | mg/day |
| Iron | mg/day |
| Magnesium | mg/day |
| Zinc | mg/day |
| Selenium | µg/day |
| Vitamin A | RE/day* |
| Vitamin C | mg/day |
| Vitamin D | µg/day |
| Vitamin E | mg/day |
| Folic Acid | µg/day |
| Beta-Carotene | µg/day |
| Caffeine | g/day |
| Garlic | g/day |
| Ginger | g/day |
| Onion | g/day |
| Saffron | g/day |
| Turmeric | mg/day |
| Pepper | g/day |
| Green/Black Tea | g/day |
